# Cases of the Cooling Treatment of Burns and Scalds

**Published:** 1806-12

**Authors:** Robert Kinglake

**Affiliations:** Taunton


### Cases of the cooling Treatment of Burns and Scalds. Communicated by Dr. Kinglake.

( Concluded from our last, pp. 460—466. )

If it were necessary to swell the list of success, recited in your last Number, it might, no doubt, be very considerably done, by the contributions which most surgical practitioners would would be able to furnish. My own experience would, indeed, supply no small addition; but I do not know that it would afford any very interesting diversity, from the experience offered in the above cases. The common accidents of this kind, cannot pretend to equal violence; and if the greater mischief is found to yield to a given remedy, the less must necessarily be under its more immediate controul.

No case has yet occurred to me, in which, the insufficiency of the cooling mode of treating burns and scalds, made it necessary to resort to any other remedy. Nor am I aware that rumour itself has attributed to its operation any suspicion even of injurious effects; much less that it should occasionally *have appeared to notice obstacles, only to be surmounted by efforts of innate energy, that could not fail to triumph over them.*

The right road to practical improvement is pursued, when reference is unceasingly had to experience. Fancy cannot impose, illusion cannot succeed, when facts, and facts alone, are made the ground of estimating the validity of a questionable opinion. In the doubt that may be still supposed to attach to the comparative virtue of *cold water,* and *heated oil of turpentine,* in curing burns and scalds, farther experience can alone avail in solving it. The subject has been already sufficiently discussed, on the respective theoretical principles that have been adduced, and now only awaits the irreversible sanction, which adequate practice can alone confer.

I am, &c.

ROBERT KINGLAKE.

*Taunton,*

*September* 23, 1806.

---

*Extract of a second Letter from Dr.* EVANS *to Dr.* KINGLAKE.

"DEAR SIR,

"I received your obliging Letter of the 2d of September, since which, I have several more cases to communicate, of the good effects of topical cold. In the first place, I will mention two, that I omitted in my former letter, which, you will of course arrange according to dates.

"Sept. 28, 1803.—Mr. Thomas Chrystle, bailiff to a colliery in this neighbourhood, belonging to John Wilkinson, Esq. had his face and hands severely burnt in a coalmine, by the explosion of hydrogen gas; cold water impregnated with a small quantity of extract of lead, was applied to the parts with the usual success. The patient being being an intelligent man, informed me that he had been burnt in coal-mines in other parts of the kingdom, and had various applications made use of, but nothing ever afforded him such immediate comfort and relief from pain, as the cold application on this occasion; to use his own words, he said "it was wonderful." On the 6th of October, he was perfectly cured with but little ulceration, the skin having separated from the face, in a dry scaly state.

"Isaac Thompson, a sinker of pits, had his face, arms, and hands, most severely burnt at the same time as Mr. Chrystle, (the subject of the above case,) but much deeper, attended with considerable tumefaction and extensive ulceration, in consequence of which, his sores were not healed till the 20th of October; but he expressed the same satisfaction, in being equally relieved from pain as his fellow sufferer, and his cure was deemed a speedy one. From a different mode of treating burns, by some practitioners in this neighbourhood, (with white-lead and oil) such injuries have been extremely tedious in their cure.

"Sept. 22, 1806.—Anthony Woollay, (an old man) and his two sons, had their faces, arms, and hands, burnt in a coal-mine, and were all immediately relieved from pain by cold applications. Large sloughs separated in a few days from the injured parts, which were dressed with the usual pledget of neutralized cerata; they are all nearly cured.

"Michael Steaton, a robust man, aged 30, on the same day, had the whole of his back burnt, he suffered the cooling treatment to be pursued till the 26th of September, when, the skin becoming loose from suppuration, a pledget of the cerate above mentioned, was applied to the extent of the injury, and repeated daily till October the 1st, when his cure was completed. He declared that he never felt pain, after 12 o'clock on the night of the accident.

"Job Fletcher, a boy, aged 12 years, had both his arms, from his shoulders to his fingers ends, deeply burnt, was treated in the same way, and with similar success, being now well.

"Sept. 22, 1806. — Thomas Ward, a young man, 19 years of age, had his feet from the instep to the toes, severely burnt by hot iron in a fluid state, as it run out of the furnace, in Ketley works. In the course of a few minutes, he was relieved from the most excruciating pain, by the cooling treatment, which he continued for the space of two days. A considerable swelling coming on, (which is common in deep burns,) a bread and milk poultice was applied to the part for a few days, in consequence of of which, a separation of the sloughs took place, and his sore is rapidly breaking by the application of the cooling cerate.

"Can there be produced by the advocates for the terebinthinate plan, more decisive evidence in favour of their stimulating mode of treatment, as to *immediate ease* and *quickness of cure?* I am so well satisfied with my successful mode of treatment, that I shall not alter it till I can find out a better. I have great opportunities for experiment. I have no other motive in making known my practice but the good of society, being arrived at that time of life, (55) in which, fame is of little consequence.

I am, Dear Sir,

Yours respectfully,

*Ketley, Shropshire,* J. EVANS."

*Oct.* 9, 1806.

